# Multicenter review of a tadalafil suspension formulation for infants and children with pulmonary hypertension: A North American experience

**DOI:** 10.3389/fped.2023.1055131

**Published:** 2023-01-26

**Authors:** David Edward Youssef, Stephanie S. Handler, Susan Marjorie Richards, Catherine Anne Sheppard, Jenna Smith, Kathryn Tillman, Matthew Pietrosanu, Edward Kirkpatrick, Angela Bates

**Affiliations:** ^1^ Division of Pediatrics, Department of Pediatric Pulmonary Hypertension, Stollery Children's Hospital, Edmonton, AB, Canada; ^2^ Division of Pediatric Cardiology, Department of Pediatrics, Medical College of Wisconsin, Milwaukee, WI, United States; ^3^ Department of Pharmacy, Stollery Children's Hospital, Edmonton, AB, Canada; ^4^ Department of Mathematical and Statistical Sciences, University of Alberta, Edmonton, AB, Canada; ^5^ Division of Pediatric Critical Care, Division of Pediatrics, University of Alberta, Edmonton, AB, Canada

**Keywords:** adverse effect, echocardiography, hemodynamic, pulmonary vasodilator, safety, tolerability

## Abstract

**Introduction:**

Phosphodiesterase type 5 (PDE5) inhibitors, with sildenafil the earliest among them, are widely used in the management of pediatric pulmonary arterial hypertension (PAH). Tadalafil is a PDE5 inhibitor with a long half life (16 h), stable pharmacokinetics and pharmacodynamics, and minimal adverse effects. However, the utility of tadalafil suspensions in this setting has not been widely explored due to a lack of clinical experience. We present a multicenter experience that details the safety and tolerability of a tadalafil suspension, either alone or in combination with another vasodilator, for the management of pediatric pulmonary hypertension (PH).

**Methods and materials:**

This is a retrospective chart review of infants and children at Children's Wisconsin and the Stollery Children's Hospital enrolled in pediatric PH programs between December 2013 and April 2022 managed with a tadalafil suspension. Patients aged six years of age and under who were treated with a tadalafil suspension were included. Demographics, clinical information, echocardiographic and hemodynamic measurements, and laboratory data were collected before and six months after tadalafil initiation.

**Results:**

Over the study period, 154 children with a median age of 1.0 (range 0.0–6.9) years were treated with tadalafil therapy. Of these, 39 (25.3%) were in group 1 (PAH), 79 (51.3%) were in group 3 (lung disease), and 33 (21.4%) were in group 5 (pulmonary hypertensive vascular disease). The median initial dose of tadalafil was 1.0 mg/kg once daily. Eleven (7.1%) patients in the cohort were established on tadalafil therapy *de novo*. The suspension formulation was necessary for 103 (66.9%) patients due to an inability to take enteral tablets and for 49 (31.8%) due to a need for feeding *via* gastric or jejunal tubes. We observed a statistically significant increase in tricuspid annular plane systolic excursion as well as significant decreases in right-ventricular systolic pressure and NT-proBNP. Tadalafil therapy was well tolerated over the six-month period: at six months, no adverse effects were reported aside from gastrointestinal disturbances by 2 (1.3%) patients.

**Conclusion:**

Tadalafil, a long-acting PDE5 inhibitor, when administered in a suspension formulation, has a safe and tolerable adverse effect profile. Following six months of therapy, our cohort showed improvements in clinical parameters, echocardiographic measurements, and laboratory results. Patient compliance was good and adverse effects were rare, minor, and manageable with nonpharmacological means.

## Introduction

Pulmonary hypertension (PH) is a progressive disease that results in right heart failure and has a high mortality among severe cases. Advancements in the treatment of pediatric PH, which have followed drug approvals for adults with pulmonary arterial hypertension (PAH), have improved quality of life and survival for pediatric patients. Therapy targeting multiple pharmacological pathways such as the nitric oxide, endothelin, and prostacyclin pathways are now available in at least four formulations (i.e., oral, inhaled, subcutaneous, and intravenous) (1–3).

All available oral therapies originated in pill form; several of these have been adapted for young pediatric patients who cannot take pills due to age or the need for administration through an enteral feeding tube. One such oral agent, the selective phosphodiesterase type 5 (PDE5) inhibitor tadalafil, was approved by the FDA for use in adults with PAH in 2009.

Tadalafil's application to adult PAH has led to significant improvements in patient exercise capacity and quality of life. Accordingly, tadalafil has since been more widely used in the pediatric population (2, 3). Tadalafil's relatively long half life and once-daily dosing facilitates improved compliance and reduced serum drug concentrations relative to sildenafil (4, 5). Compound tadalafil suspensions have permitted further flexibility with regard to dosing and administration in infants and children.

However, experiences with tadalafil suspensions in monotherapy or combination therapy for pediatric PH are not widely available in the literature. The purpose of this study is to describe the use of an enteral tadalafil suspension in two large North American pediatric PH centers. Specifically, our primary goal is to describe the safety and tolerability of tadalafil suspensions for infants and children six years of age and under with PH. Our secondary goal is to demonstrate the safety and clinical utility of transitioning from enteral sildenafil to an enteral tadalafil suspension that has previously been presented in the literature (6).

## Materials and methods

This is a retrospective, descriptive, two-center study analyzing clinical and echocardiographic data collected from infants and children six years of age and under enrolled in the pediatric PH programs at the Stollery Children's Hospital from January 1, 2014 to April 30, 2022 and at Children's Wisconsin from December 11, 2013 to March 8, 2022 and managed with a tadalafil suspension. We collected data on patient demographics, clinical characteristics, PH diagnoses following the 6th World Symposium on Pulmonary Hypertension (WSPH6) (directly from clinical notes), tadalafil dosing, prior and concurrent treatments, adverse effects and treatment tolerability, and echocardiographic parameters based on center-specific protocols and biochemical measures before and six months following tadalafil initiation. Children's Wisconsin was not able to provide some echocardiographic parameters.

Echocardiographic parameters were obtained under the institutions' pediatric PH protocols. Imaging was obtained from subcostal, apical 4 chamber, parasternal long-axis, and parasternal short-axis views. Information on shunt size and direction was retrieved wherever possible. Right-ventricular systolic pressure was calculated as the sum of tricuspid regurgitation velocity and right-atrial pressure. Eccentricity index was calculated from the parasternal short-axis view as the ratio between the major and minor dimensions of the left ventricle at end systole and at the papillary muscle level. Right-ventricular fractional area change in systole and diastole at the basal, papillary, and apical levels were calculated from an optimized right ventricle view. Right–left ventricle diameter ratio was obtained at end systole. Mean pulmonary arterial pressure was estimated from end-diastolic pulmonary insufficiency. All patients were treated under the same standardized PH protocol at both institutions. Patients were not included if they were not treated with a tadalafil suspension as part of their treatment strategy.

Ethics approvals were obtained from the Health Research Ethics Board at the University of Alberta (Pro00120489) and the Institutional Review Board at Children's Wisconsin (1704191-3).

Statistical analyses were performed using R version 3.6.3 (7). Categorical data are summarized with counts and percentages, and numeric variables with the first through third quartiles. Paired pre–post comparisons were conducted using paired Wilcoxon signed rank tests; corresponding *p*-values are adjusted (across the main text) with a Benjamini–Hochberg correction for false discovery. We use a threshold significance of 0.05 throughout.

## Results

### Patient cohort

**Figure 1:**
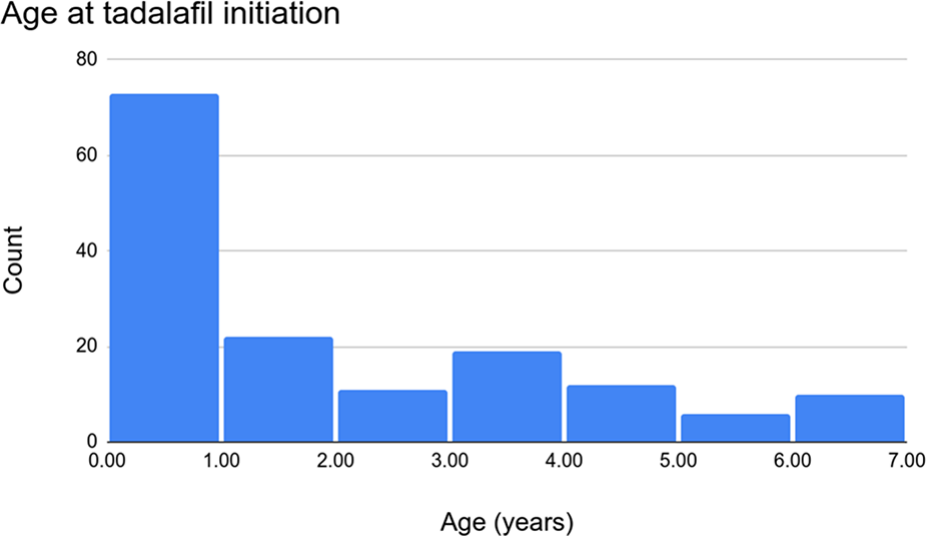
Histogram of age at tadalafil initiation.

Over the study period, 154 patients were treated with tadalafil. Of these, 72 (46.8%) were female. The median age at the initiation of tadalafil therapy was 1.0 (range 0.0–6.9) years (Figure 1) with a median BMI of 15.3 (Q_1_ = 14.2, Q_3_ = 16.8) kg/m^2^. Of the 154 patients, 4 (2.6%) went on to require transplantation and 146 (94.8%) were alive at a six-month evaluation. In terms of ethnicity, 99 (64.3%) patients were Caucasian, 23 (14.9%) were Black, 10 (6.5%) were Indigenous, and 10 (6.5%) were Asian. Trisomy 21 was documented in 21 (13.6%) patients, DiGeorge syndrome in 3 (1.9%), and trisomy 18 in 4 (2.6%). See Table 1 for more detail regarding the demographics, anthropometrics, and genetic conditions of our cohort.

**Table 1:** Patient demographic and clinical characteristics at tadalafil initiation, summarized as median (Q_1_, Q_3_) or count (%).

Variable/level	Summary
**Demographics/anthropometry**	
Sex: female	72 (46.8%)
Age (years)	1.0 (0.5, 3.1)
Weight (kg)	7.9 (5.7, 13.0)
Height (cm)	71.8 (59.9, 94.2)
BMI (kg/m^2^)	15.3 (14.2, 16.8)
**Ethnicity**	
Asian	10 (6.5%)
Black	23 (14.9%)
Caucasian	99 (64.3%)
Indigenous	10 (6.5%)
Other	12 (7.8%)
**Genetic syndromes**	
DiGeorge syndrome	3 (1.9%)
Noonan syndrome	3 (1.9%)
Trisomy 18	4 (2.6%)
Trisomy 21	21 (13.6%)
Other	9 (5.8%)
**WSPH6 classification**	
Group 1	39 (25.3%)
Group 2	1 (0.6%)
Group 3	79 (51.3%)
Group 4	1 (0.6%)
Group 5	33 (21.4%)

Under the WSPH6 diagnostic classification, 79 patients (51.3%) were in group 3, 39 (25.3%) were in group 1, 33 (21.4%) were in group 5, 1 (0.6%) was in group 2, and 1 (0.6%) was in group 4.

### Effect of tadalafil on functional capacity

**Figure 2:**
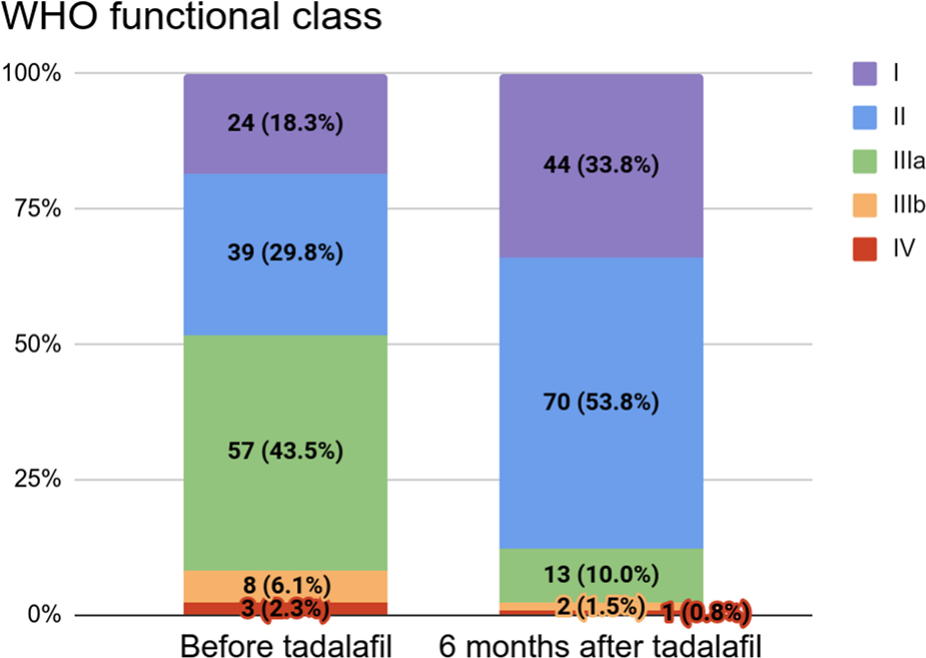
WHO functional class before (*n* = 131) and six months after (*n* = 130) tadalafil initiation.

Among the 131 patients with a WHO functional class (WHO FC) available at baseline, 57 (43.5%) were in class IIIa, 39 (29.8%) were in class II, 24 (18.3%) were in class I, 8 (6.1%) were in class IIIb, and 3 (2.3%) were in class IV. Among these patients, 130 had a WHO FC available six months after tadalafil initiation: 70 (53.8%) were in class II, 44 (33.8%) were in class I, 13 (10.0%) were in class IIIa, 2 (1.5%) were in class IIIb, and 1 (0.8%) was in class IV (Supplementary Table S1). These distributions are shown in Figure 2.

### Tadalafil dosing and adverse effects

The majority of patients were transitioned from sildenafil to tadalafil; only 11 (7.1%) patients started liquid tadalafil *de novo*. The latter were given lower doses (0.25–0.50 mg/kg) for one–seven days depending on side effect tolerability and inpatient status. Doses were then increased every two–three days (to 1.0 mg/kg for patients under 25 kg, 20.0 mg for patients between 25 kg and 40 kg, and 40.0 mg for patients over 40 kg). The maximum dose utilized was 40.0 mg once daily. Patients who were transitioned from sildenafil to tadalafil had a direct conversion to a full 1.0 mg/kg daily dose (up to a maximum dose of 40.0 mg once daily) that replaced the next dose of sildenafil. Sildenafil was subsequently discontinued.

Table 2 describes the cohort's indications for a tadalafil suspension, tadalafil dosing, prior treatment with sildenafil, and additional therapies. At initiation, the median dose of tadalafil was 1.0 (range 0.3–1.3) mg/kg once daily. About two-thirds of patients required a tadalafil suspension due an inability to swallow tablets (stemming from age). The median duration of sildenafil therapy prior to tadalafil was 30.0 (Q_1_ = 3.0, Q_3_ = 192.5) days. At initiation, 26 (16.9%) patients were additionally being treated with bosentan, 16 (10.4%) with ambrisentan, and 1 (0.6%) with macitentan.

**Table 2:** Patient medication, summarized as median (minimum–maximum), median (Q_1_, Q_3_), or count (%).

Variable/level	Summary
**Tadalafil dosing**	
Initial dose (mg/kg)	1.0 (0.3–1.3)
Maximum dose^a^ (mg/kg)	1.0 (0.5–1.0)
Time to maximum dose^a^ (days)	1.0 (0.0–766.0)
**Indication for tadalafil suspension** ^b^	
Age (unable to take oral tablets)	103 (66.9%)
Tube fed (gastric or jejunal tube feeds)	49 (31.8%)
**Prior sildenafil treatment** ^b^	
Prior sildenafil duration (days)	30.0 (3.0, 192.5)
**Additional vasodilator medical therapy** ^b^	
Ambrisentan	16 (10.4%)
Bosentan	26 (16.9%)
Macitentan	1 (0.6%)

^a^ The values of Q_1_ and Q_3_ for these variables are all 1.0 mg/kg or 1.0 days, with the exception of time to maximum dose, which has Q_3_ = 2.0 days.

^b^ At tadalafil initiation.

Of the few adverse treatment effects reported before initiation, reflux was noted in 4 patients (2.6%), flushing or rashes in 2 (1.3%), and other side effects in 5 (3.2%) (namely, desaturation and ventilation/perfusion mismatch, hair thinning/loss, pulmonary edema, and urinary incontinence). After six months of tadalafil therapy, only 2 (1.3%) patients reported side effects (specifically, reflux) and 146 (94.8%) patients reported no significant adverse effects. No obvious differences in side effects between the transition and *de novo* groups (notably, with respect to hypotension, dizziness, and gastrointestinal symptoms) were observed. See Table 3 for a summary of adverse effects at both time points.

**Table 3:** Adverse effects of treatment before and six months after tadalafil initiation, summarized as count (%).

Side effect	Before tadalafil	Six months after tadalafil
Dermatological^a^	2 (1.3%)	0 (0.0%)
Ears, nose, and throat	0 (0.0%)	0 (0.0%)
Gastrointestinal^b^	4 (2.6%)	2 (1.3%)
Hypotension	0 (0.0%)	0 (0.0%)
Neurological	1 (0.6%)	0 (0.0%)
Other^c^	5 (3.2%)	0 (0.0%)
None	143 (92.9%)	146 (94.8%)

^a^ Dermatological adverse effects (before tadalafil) included one case of flushing and one case of rashing.

^b^ Gastrointestinal adverse effects consisted entirely of reflux (gagging or retching).

^c^ Other symptoms included frequent desaturation and ventilation/perfusion mismatch, hair thinning/loss, pulmonary edema, and urinary incontinence. One “other” symptom was not reported.

### Effects of tadalafil on echocardiographic measures and biochemistry

Changes in echocardiographic parameters from baseline (prior to tadalafil) to six months after initiation are summarized in Table 4. Median right-ventricular systolic pressure (RVSP) decreased over the six-month period from 50.5 (Q_1_ = 35.0, Q_3_ = 64.0) mmHg to 37.0 (Q_1_ = 30.0, Q_3_ = 48.5) mmHg. There was no appreciable change in median mean pulmonary arterial pressure (mPAP) from 15.3 (Q_1_ = 6.1, Q_3_ = 29.5) mmHg to 17.0 (Q_1_ = 10.0, Q_3_ = 33.0) mmHg. Although we observed a slight improvement in right-ventricular fractional area change (RVFAC) from 35.0% (Q_1_ = 30.3%, Q_3_ = 42.8%) to 37.1% (Q_1_ = 31.0%, Q_3_ = 44.0%), the median change per patient was negative at −0.9 (Q_1_ = −5.3, Q_3_ = 8.0) mmHg. Median tricuspid annular plane systolic excursion (TAPSE) improved slightly from 1.1 (Q_1_ = 0.8, Q_3_ = 1.6) cm to 1.3 (Q_1_ = 1.1, Q_3_ = 1.7) cm and median eccentricity index decreased slightly from 1.3 (Q_1_ = 1.1, Q_3_ = 1.7) to 1.2 (Q_1_ = 1.0, Q_3_ = 1.4). Of these changes, only those for RVSP and TAPSE were statistically significant (*p* ≤ 0.003).

**Table 4:** Echocardiographic measures before and six months after tadalafil initiation, summarized as median (Q_1_, Q_3_) or count (%).

Variable/level	Before tadalafil	Six months after tadalafil	Difference^a^
**Echocardiographic parameters**			
Eccentricity index	1.3 (1.1, 1.7)	1.2 (1.0, 1.4)	−1.0 (−0.4, 0.1) [*p* = 0.083]
mPAP (mmHg)	15.3 (6.1, 29.5)	17.0 (10.0, 33.0)	0.5 (−1.3, 2.0) [*p* = 0.953]
RV/LV	1.0 (0.8, 1.3)	0.7 (0.6, 1.0)	−0.2 (−0.5, 0.1) [*p* = 0.064]
RVFAC (%)	35.0 (30.3, 42.8)	37.1 (31.0, 44.0)	−0.9 (−5.3, 8.0) [*p* = 0.929]
RVSP (mmHg)	50.5 (35.0, 64.0)	37.0 (30.0, 48.5)	−8.0 (−20.0, 0.8) [*p* = 0.003]
TAPSE (cm)	1.1 (0.8, 1.6)	1.3 (1.1, 1.7)	0.3 (0.0, 0.5) [*p* = 0.001]
**Atrial septal defect shunt**			
Presence of shunt	77 (50.0%)	60 (39.0%)	
Shunt direction: left to right	44 (28.6%)	38 (24.7%)	
Shunt direction: right to left	4 (2.6%)	4 (2.6%)	
Shunt direction: bidirectional	25 (16.2%)	16 (10.4%)	
Shunt gradient (mmHg)	2.3 (1.8, 7.4)	3.9 (1.6, 4.9)	0.0 (−2.3, 0.0)
**Ventricular septal defect shunt**			
Presence of shunt	26 (16.9%)	23 (14.9%)	
Shunt direction: left to right	17 (11.0%)	15 (9.7%)	
Shunt direction: right to left	3 (1.9%)	2 (1.3%)	
Shunt direction: bidirectional	6 (3.9%)	5 (3.2%)	
Shunt gradient (mmHg)	36.0 (33.0, 39.0)	36.0 (28.5, 42.5)	5.0 (3.0, 7.5)

Note. RV/LV, right–left ventricle diameter ratio.

^a^ The Difference column reports paired pre–post differences: *n* = 12 for mPAP, *n* ≤ 7 for the shunt gradients, and *n* ≥ 45 for all other variables. Formal testing is omitted for both shunt gradients due to the small sample size.

Atrial septal defects (ASDs) were present in 77 (50.0%) patients at baseline: of these, 44 had a left-to-right shunt and 25 had a bidirectional shunt. At six months, 60 (39.0%) patients had an ASD: among these, 38 had a left-to-right shunt and 16 had a bidirectional shunt. Ventricular septal defects (VSDs) were present in 26 (16.9%) patients at baseline: of these, 17 had left-to-right shunts and 6 had bidirectional shunts. Following six months of therapy, 23 (14.9%) patients had a VSD: among these, 15 had a left-to-right shunt and 5 had a bidirectional shunt.

Table 5 summarizes biochemical measures for the cohort. We observed a statistically significant change (*p* = 0.001) in median NT-proBNP levels from 659.0 (Q_1_ = 217.0, Q_3_ = 2261.0) ng/L to 346.0 (Q_1_ = 194.5, Q_3_ = 1125.8) ng/L from baseline to six months after initiation. Statistically significant changes in other biochemical measures (e.g., hemoglobin, urea, creatinine, aspartate transaminase, and alanine transaminase) were not detected (*p* ≥ 0.064).

**Table 5:** Biochemical measures before and Six months after tadalafil, summarized as median (Q_1_, Q_3_).

Variable	Before tadalafil	Six months after tadalafil	Difference^a^
ALT (units/L)	21.0 (17.0, 34.5)	21.5 (15.3, 34.8)	−2.0 (−9.0, 6.0) [*p* = 0.241]
AST (units/L)	37.0 (28.0, 48.8)	36.5 (27.0, 47.8)	−2.0 (−14.3, 6.0) [*p* = 0.083]
Creatinine (umol/L)	27.0 (21.0, 35.0)	24.0 (20.0, 33.0)	−1.0 (−6.0, 4.0) [*p* = 0.240]
Hgb (g/L)	127.0 (112.5, 140.0)	126.0 (113.3, 135.8)	−1.0 (−18.5, 10.5) [*p* = 0.330]
NT-proBNP (ng/L)	659.0 (217.0, 2261.0)	346.0 (194.5, 1125.8)	−372.0 (−1408.0, 31.5) [*p* = 0.001]
Total bilirubin (umol/L)	9.0 (6.3, 16.0)	8.0 (5.0, 12.0)	−2.0 (−7.0, 2.0) [*p* = 0.064]
Urea (mmol/L)	5.0 (3.7, 7.0)	4.0 (3.0, 6.1)	−0.2 (−2.0, 1.2) [*p* = 0.330]
WBC (10^9^/L)	8.9 (7.0, 11.6)	8.2 (6.6, 11.3)	−0.3 (−3.8, 1.4) [*p* = 0.360]

Note. AST, aspartate transaminase; ALT, alanine transaminase; Hgb, hemoglobin; NT-proBNP, N-terminal pro B-natriuretic peptide; WBC, white blood cell count.

^a^ The Difference column reports paired pre–post differences as median (Q_1_, Q_3_) [*p*-value]: *n* ≥ 77 for all variables.

### Mortality

In our cohort of 154 patients, there were a total of 8 (5.2%) deaths in the six months following initiation. Of these deaths, 5 were male. The patients who died were aged 0–15 years. None of the deaths were directly related to tadalafil and none featured severe side effects. Two patients had progressive severe pulmonary vein stenosis (PVS) with an element of precapillary PH and were initiated on the tadalafil suspension and followed closely. Three patients had progressive ventricular dysfunction and were not candidates for heart or lung transplants (1 severe left-ventricular diastolic dysfunction, 1 failing Glenn, 1 failing Fontan); these patients remained on tadalafil for symptom improvement. Neither the patients with progressive ventricular dysfunction nor the PVS patients had significant pulmonary edema secondary to Tadalafil. Two patients had severe developmental lung disease (1 bronchopulmonary dysplasia, 1 alveolar capillary dysplasia). Both of these patients were too severe to survive to a transplant work-up/listing and died despite additional aggressive PAH therapy. One patient received tadalafil for severe idiopathic PAH in a liquid formulation while intubated and died following a lung transplant from severe end-organ dysfunction; this patient was off liquid tadalafil following the transplant. All patients in this group tolerated the suspension formulation of tadalafil. All were intubated and required a liquid formulation as a method of administration *via* a feeding tube, including the older patients.

### Comparisons between PH groups

Supplementary Tables S2–S4 present comparisons of hemodynamic and echocardiographic parameters, biochemistry levels, and adverse effects between the patients in groups 1, 3, and 5. In comparing echocardiographic parameters, we focus on groups 1 and 3 here since these parameters cannot be meaningfully interpreted for complex single-ventricle patients. There were no significant differences detected in pre–post changes between the groups. We observed clinically significant differences in NT-proBNP between groups 1 and 3 both before and six months after tadalafil initiation. The median NP-proNBP among group 1 patients was 659.0 (Q_1_ = 199.0, Q_3_ = 2464.0) ng/L at baseline and 255.0 (Q_1_ = 136.0, Q_3_ = 975.0) ng/L after six months. For group 3 patients, this was 426.0 (Q_1_ = 217.0, Q_3_ = 1481.5) ng/L at baseline and 381.0 (Q_1_ = 199.0, Q_3_ = 915.0) ng/L after six months.

## Discussion

### The role of PDE5 inhibitors

The lack of randomized clinical trials in the context of pediatric PAH has led to a dependence on consensus statements and recommendations when managing children with the condition (8). Previous systematic reviews have highlighted the relatively small number of randomized controlled trials using PDE5 inhibitors such as tadalafil to treat pediatric patients with PAH (8). Despite the beneficial effects of this therapy in improving oxygenation, hemodynamics, and exercise capacity (1–5, 8), some centers have not transitioned from multiple to once-daily PDE5 inhibitor dosing. This may be due to center preference and further driven by a lack of multicenter data and experience, particularly with pediatric patients. For this reason, we presented our experience regarding the safety and tolerability of once-daily enteral tadalafil dosing and the process of transitioning from sildenafil to tadalafil.

There exists a growing body of evidence supporting the use of dual therapy to treat PAH (where PDE5 inhibitors play a crucial role), either alone or in combination treatment strategies (9). Consensus statements have reiterated the importance of PDE5 inhibitors in treating pediatric patients at any stage (10). Although early pediatric guidelines and the literature focused on sildenafil, suspensions for this drug administered thrice daily have the potential to contribute to poor compliance and missed doses in the pediatric population (10–12). The relative ease of administering a tadalafil suspension enterally (e.g., for the 31.8% of patients in the present study who required tube feeding or for infants with comorbidities) along with its long, 90-day shelf life (6) reduces the risk of inaccurate dosing on the part of caregivers and physicians.

### Tadalafil transition and dosing

The preference of the two institutions in this study is for patients established on a thrice-daily 1.0 mg/kg dose of sildenafil to be transitioned to a once-daily 1.0 mg/kg dose of tadalafil. The tadalafil suspension used at the centers in this study has been described in other studies (6). We have found this suspension to be stable, palatable, and able to be stored for prolonged periods of time. Future work should consider starting tadalafil *de novo* in neonates and children and target doses of 1.0 mg/kg once daily.

All previous single-center descriptive experiences with tadalafil in the pediatric population (2–4, 13) included patients on sildenafil for at least six months prior to a transition to tadalafil. Our work uniquely highlighted the possibility of a prompt transition from thrice-daily sildenafil to once-daily tadalafil. In our cohort, the median time to transition from full-dose sildenafil to the tadalafil equivalent was one day. Among the 154 patients, 11 (7.1%) were treated with tadalafil *de novo*, i.e., with no prior transition.

### Safety and clinical changes

A previous review of the efficacy and safety of PDE5 inhibitor therapy examined the potential adverse effects of tadalafil (13). This prospective, open-label study included 25 patients between the ages of 0.2 and 5.0 years who were initially treated with 1.0 mg/kg of tadalafil once daily. Six of the 25 patients were previously treated with sildenafil. The authors reported that 3 (12.0%) patients experienced headaches and flushing, but that tadalafil was otherwise well tolerated by the cohort (13). In the present study, the safety and tolerability of the therapy was evident as only a few patients experienced adverse effects. At baseline, 4 (2.6%) patients had gastrointestinal side effects, 2 (1.3%) had either flushing or rashes, and 1 (0.6%) had neurological side effects. Six months after initiation, however, only 2 (1.3%) patients reported adverse effects (namely, gastrointestinal). All adverse effects were manageable with non-pharmacological means (e.g., a change in the time of administration or medicating with meals). The patients who started tadalafil *de novo* did not have any adverse effects, which is promising as an initial result. No patients required hospitalization or a discontinuation of therapy. We hypothesize that differences in the adverse effects reported in this study relative to previous works can be attributed to our tendency to establish infants and neonates on a thrice-daily 1.0 mg/kg dose of sildenafil prior to transitioning to a once-daily 1.0 mg/kg dose of tadalafil.

Over the study period, we observed clinical improvements in hemodynamic, biochemical, and echocardiographic measures and statistically significant changes in NT-proBNP levels, RVFAC, and TAPSE. These changes were consistent with the observed changes in WHO FC. However, given the large number of patients in groups 1 and 3, improvements may have been expected and might not be directly correlated to tadalafil usage, especially in comparison to historical therapies such as sildenafil.

In early experiences, the immature glucuronidation pathways of the neonatal and infant populations seemed to contraindicate tadalafil (10). However, pharmacokinetic-based modeling for children under two years of age has established that once-daily tadalafil is safe and efficacious in the pediatric PAH population (9, 14). The current work, even with its small number of *de novo* patients, highlighted the potential for future treatment strategies for pediatric PAH.

Since the 2018 World Symposium on Pulmonary Hypertension, single-ventricle patients with cavopulmonary anastomosis have been reclassified into group 5 (15). This is in addition to a growing population of complex single-ventricle patients who are palliated to Fontan circulation. In this cohort, patients with group 5 pulmonary hypertensive vascular disease (PHVD) made up a startling 21.4% of the patients receiving liquid tadalafil. This study was neither designed nor powered to look more closely at this group of complex patients. We recognize the limited literature on how PDE5 inhibitors should be used in this population. Based on the present study, liquid tadalafil appears to be a feasible, well-tolerated medication for single-ventricle patients meeting the criteria for PHVD.

### Comparisons between groups 1, 3, and 5

While the focus of this paper was predominantly on the tolerance and safety of a tadalafil suspension for pediatric PH patients, we also compared patients across groups 1, 3, and 5. Our inclusion of pediatric PH patients in these three groups also makes the current study unique. Some previous studies on tadalafil therapy were limited to group 1 patients (3, 4, 13) the current research does not have this restriction and thus presents a broader look at tadalafil as a treatment for pediatric PAH and highlights the general tolerability of this therapy.

Side effects–including commonly reported ones such as gastrointestinal disturbances, congestion, and hypotension–were rare in these groups after six months of tadalafil (Supplementary Table S2). This may be due to the large number of patients who were already established on sildenafil prior to transitioning to the tadalafil suspension or it may be that many of the side effects occurred initially but resolved with time. While median RVFAC decreased overall, this may be due to differences in measurement, as has been reported in the literature (16). Group 3 patients showed clinical improvements in terms of eccentricity index, right–left ventricle diameter ratio, RVFAC, RVSP, and TAPSE (Supplementary Table S3). Since many of these patients have PH associated with bronchopulmonary disease and would have grown significantly over the six-month period, the observed increase in mPAP (to normal levels of under 20.0 mmHg) might have been expected.

In comparing changes in biochemistry across the three groups (Supplementary Table S4), prior to starting suspension tadalafil and six months after therapy, the majority of the markers would be related to side effects, of which liver, kidney and bone marrow changes were not clinically significant. NT-proBNP in both groups 1 and 3 saw a clinically significant decline to normal levels after six months of the tadalafil suspension. For the group 1 patients where tadalafil was targeting the pulmonary arterial bed, this improvement is expected since many of these patients have postoperative-associated PAH. Among group 3 patients, for whom the literature is still evolving with respect to the role of pulmonary vasodilators, it is encouraging to see an improvement in NT-proBNP that coincides with clinical improvement. It is impossible to know whether pulmonary vascular bed growth over time or the tadalafil suspension had a larger impact on this biochemical marker. More research is needed in this area.

Among group 5 patients, who were largely complex single-ventricle patients following Fontan palliative surgery, it was not surprising to see a trend toward increasing values of NT-proBNP. It is often struggling single-ventricle patients who are considered for pulmonary vasodilator therapy—first for PDE5 inhibitors. However, it is not known whether the management of PHVD has an effect on outcomes in this population or if PHVD management is a marker of a poor prognosis. More research needs to be done specifically in this growing group of patients to determine an approach to pulmonary vasodilator therapy that includes the use of tadalafil.

### Limitations

The present work has a number of limitations. First, while it would be ideal to have data from right-heart catheterization and additional data from magnetic resonance imaging, this is not practical given the age of the patients and the need for anesthesia. We have instead used noninvasive bedside measurements. Second, WHO FC as an instrument for measuring response to therapy has not been validated among infants and children. Similarly, the six-minute walk test, while appropriate in studies of adults, is not applicable or reproducible within the current cohort. Future work examining the long-term pharmacokinetics of PDE5 inhibitors such as tadalafil is necessary as relevant data in the present study is lacking.

## Conclusion

Our descriptive analysis of a heterogeneous group of PH patients highlights the tolerability and safety of tadalafil suspensions for infants and children six years of age and under. Tadalafil suspensions administered once daily have a safe and efficacious medication profile for pediatric PH patients. We observed clinically significant medium-term improvements in clinical, echocardiographic, and laboratory parameters among pediatric PH patients treated with a tadalafil suspension, both alone and in combination with other treatments.

## Data Availability

The datasets presented in this article are not readily available due to ethics restrictions. Requests to access the datasets should be directed to the corresponding author.
