# Supplementary material for: Multicenter review of a tadalafil suspension formulation for infants and children with pulmonary hypertension: A North American experience
Source: Front Pediatr. 2023 Jan 26;11:1055131. doi: 10.3389/fped.2023.1055131 (PMC9909267; doi:10.3389/fped.2023.1055131)
Supplement: Supplementary file 1 [file Datasheet1.pdf]

Supplementary Material

1 Supplementary Tables

Supplementary Table 1. WHO Functional Class Before and Six Months After Tadalafil Initiation, Summarized as Count (%)

**Class**	**Before tadalafil** (n = 131)	**Six months after tadalafil** (n = 130)
Class I	24 (18.3%)	44 (33.8%)
Class II	39 (29.8%)	70 (53.8%)
Class IIIa	57 (43.5%)	13 (10.0%)
Class IIIb	8 (6.1%)	2 (1.5%)
Class IV	3 (2.3%)	1 (0.8%)

Supplementary Table 2. Adverse Effects of Treatment Before and Six Months After Tadalafil Initiation, Summarized as Count (%) and Stratified by WSPH6 Classification at Initiation

**Side effect**	**Before tadalafil**			**Six months after tadalafil**		
	Group 1 (n = 39)	Group 3 (n = 79)	Group 5 (n = 33)	Group 1 (n = 37)	Group 3 (n = 76)	Group 5 (n = 32)
Dermatological[1]	1 (2.6%)	0 (0.0%)	1 (3.0%)	0 (0.0%)	0 (0.0%)	0 (0.0%)
Ears, nose, and throat	0 (0.0%)	0 (0.0%)	0 (0.0%)	0 (0.0%)	0 (0.0%)	0 (0.0%)
Gastrointestinal[2]	2 (5.1%)	1 (1.3%)	1 (3.0%)	1 (2.7%)	1 (1.3%)	0 (0.0%)
Hypotension	0 (0.0%)	0 (0.0%)	0 (0.0%)	0 (0.0%)	0 (0.0%)	0 (0.0%)
Neurological	0 (0.0%)	0 (0.0%)	1 (3.0%)	0 (0.0%)	0 (0.0%)	0 (0.0%)
Other[3]	1 (2.6%)	2 (2.5%)	1 (3.0%)	0 (0.0%)	0 (0.0%)	0 (0.0%)
None	35 (89.7%)	76 (96.2%)	30 (90.9%)	36 (97.3%)	75 (98.7%)	32 (100.0%)

[1] Dermatological adverse effects (before tadalafil) included one case of flushing and one case of rashing.

[2] Gastrointestinal adverse effects consisted entirely of reflux (gagging or retching).

[3] Other symptoms included frequent desaturation and ventilation/perfusion mismatch (group 3), hair thinning/loss (group 1), pulmonary edema (group 3), and urinary incontinence (group 5).

Supplementary Table 3. Echocardiographic Measures Before and Six Months After Tadalafil Initiation, Summarized as Median (Q_1, Q_3) [*n*] and Stratified by WSPH6 Classification at Initiation

**Variable**	**Before tadalafil**			**Six months after tadalafil**			**Difference**[1]
	Group 1	Group 3	Group 5	Group 1	Group 3	Group 5	
Eccentricity index	1.3 (1.1, 1.6) [*n* = 15]	1.3 (1.1, 1.6) [*n* = 31]	1.9 (1.5, 2.0) [*n* = 3]	1.2 (1.0, 1.4) [*n* = 15]	1.2 (1.0, 1.5) [*n* = 34]	1.0 (0.9, 1.4) [*n* = 7]	0.744* [*n* = 42]
mPAP (mmHg)	16.5 (8.3, 34.3) [*n* = 8]	13.0 (5.3, 21.0) [*n* = 14]	20.2 (12.0, 27.0) [*n* = 5]	11.5 (9.5, 33.3) [*n* = 8]	18.0 (10.5, 34.5) [*n* = 11]	17.0 (17.0, 17.0) [*n* = 1]	0.137* [*n* = 11]
RV/LV ratio	1.1 (0.7, 1.4) [*n* = 15]	0.9 (0.7, 1.1) [*n* = 33]	1.1 (1.0, 1.6) [*n* = 4]	0.9 (0.6, 1.1) [*n* = 15]	0.7 (0.6, 0.9) [*n* = 36]	0.5 (0.5, 1.6) [*n* = 6]	0.495* [*n* = 43]
RVFAC (%)	39.0 (28.8, 45.8) [*n* = 20]	33.5 (29.8, 42.0) [*n* = 36]	35.2 (34.0, 39.3) [*n* = 8]	29.0 (31.5, 46.6) [*n* = 19]	37.0 (30.0, 44.0) [*n* = 33]	34.0 (32.0, 39.0) [*n* = 13]	0.394 [*n* = 55]
RVSP (mmHg)	49.5 (36.3, 63.0) [*n* = 26]	50.0 (35.0, 65.0) [*n* = 44]	68.0 (46.0, 89.5) [*n* = 4]	36.0 (31.5, 50.0) [*n* = 20]	38.0 (30.0, 46.0) [*n* = 45]	30.0 (28.5, 65.3) [*n* = 6]	0.240* [*n* = 52]
TAPSE (cm)	1.1 (0.9, 1.7) [*n* = 21]	1.1 (0.8, 1.7) [*n* = 43]	0.9 (0.8, 1.0) [*n* = 8]	1.3 (1.0, 1.8) [*n* = 23]	1.4 (1.2, 1.8) [*n* = 46]	1.2 (1.1, 1.4) [*n* = 12]	0.737 [*n* = 56]

Note. mPAP, mean pulmonary arterial pressure; RV/LV ratio, ratio of right to left ventricle diameter; RVFAC, right-ventricular fractional area change; RVSP, right-ventricular systolic pressure; TAPSE, tricuspid annular plane systolic excursion

[1] The Difference column reports unadjusted p-values for a comparison of paired pre–post differences between the groups conducted via Kruskal–Wallis tests. An asterisk denotes comparisons where group 5 was excluded for having a small sample size ($n < 5$).

Supplementary Table 4. Biochemical Measures Before and Six Months After Tadalafil, Summarized as Median (Q_1, Q_3) [*n*] and Stratified by WSPH6 Classification at Initiation

**Variable**	**Before tadalafil**			**Six months after tadalafil**			**Difference**[1]
	Group 1	Group 3	Group 5	Group 1	Group 3	Group 5	
ALT (units/L)	20.0 (18.0, 34.0) [*n* = 31]	22.5 (18.0, 38.5) [*n* = 58]	17.0 (14.8, 24.3) [*n* = 24]	22.0 (13.0, 25.8) [*n* = 28]	23.0 (16.0, 35.3) [*n* = 48]	20.0 (16.5, 44.0) [*n* = 16]	0.420 [*n* = 85]
AST (units/L)	39.0 (28.5, 50.5) [*n* = 31]	37.0 (28.0, 46.0) [*n* = 57]	37.0 (28.0, 45.8) [*n* = 24]	35.0 (28.0, 39.0) [*n* = 27]	38.0 (27.0, 51.5) [*n* = 47]	38.0 (31.5, 51.5) [*n* = 16]	0.322 [*n* = 82]
Creatinine (umol/L)	32.0 (25.0, 35.5) [*n* = 35]	24.0 (19.5, 28.0) [*n* = 69]	29.0 (25.0, 37.0) [*n* = 29]	29.0 (20.0, 38.0) [*n* = 31]	22.0 (18.0, 27.0) [*n* = 60]	30.0 (26.3, 34.5) [*n* = 22]	0.817 [*n* = 113]
Hgb (g/L)	132.0 (118.5, 144.5) [*n* = 34]	124.0 (110.0, 134.0) [*n* = 57]	127.5 (117.3, 151.8) [*n* = 26]	132.0 (111.0, 136.0) [*n* = 29]	123.5 (113.3, 133.0) [*n* = 58]	128.0 (117.0, 145.0) [*n* = 21]	0.104 [*n* = 97]
NT-proBNP (ng/L)	659.0 (199.0, 2464.0) [*n* = 25]	426.0 (217.0, 1481.5) [*n* = 52]	1910.0 (341.0, 5190.0) [*n* = 13]	255.0 (136.0, 975.0) [*n* = 21]	381.0 (199.0, 915.0) [*n* = 41]	2100.0 (373.0, 2498.5) [*n* = 7]	0.178 [*n* = 59]
Total bilirubin (umol/L)	9.5 (7.0, 12.5) [*n* = 30]	7.0 (5.0, 14.5) [*n* = 58]	11.0 (7.0, 22.0) [*n* = 24]	5.5 (4.0, 8.0) [*n* = 26]	9.0 (6.0, 12.0) [*n* = 45]	8.5 (5.0, 13.3) [*n* = 14]	0.257 [*n* = 76]
Urea[2] (mmol/L)	5.0 (3.5, 6.1) [*n* = 35]	5.0 (3.5, 7.0) [*n* = 70]	5.6 (4.1, 7.5) [*n* = 28]	4.9 (3.6, 6.7) [*n* = 31]	4.0 (2.8, 5.9) [*n* = 58]	4.2 (2.6, 5.4) [*n* = 22]	0.046 [*n* = 110]
WBC (10^9/L)	8.2 (6.1, 11.0) [*n* = 34]	9.4 (7.9, 11.7) [*n* = 57]	8.6 (6.9, 9.8) [*n* = 26]	6.9 (6.0, 9.2) [*n* = 29]	9.7 (7.7, 11.7) [*n* = 57]	6.9 (4.6, 9.4) [*n* = 21]	0.442 [*n* = 96]

Note. AST, aspartate transaminase; ALT, alanine transaminase; Hgb, hemoglobin; NT-proBNP, N-terminal pro B-natriuretic peptide; WBC, white blood cell count

[1] The Difference column reports unadjusted *p*-values for a comparison of paired pre–post differences between the groups.

[2] Unadjusted post hoc comparisons of Urea between groups with Dunn's test yields unadjusted *p*-values of 0.143 (group 1 vs. group 3), 0.050 (group 1 vs. group 5), and 0.279 (group 3 vs. group 5). The median (Q_1, Q_3) pre–post difference in Urea for each group was 0.60 (-1.0, 1.9) for group 1 (n = 31), -0.6 (-2.3, 1.2) for group 3 (n = 58), and -0.8 (-2.7, 0.3) for group 5 (n = 21).
